# Incident atrial fibrillation in patients with differentiated thyroid cancer: a meta-analysis

**DOI:** 10.1530/ERC-20-0496

**Published:** 2021-03-31

**Authors:** Georgios Kostopoulos, Ioannis Doundoulakis, Christina Antza, Emmanouil Bouras, Krishnarajah Nirantharakumar, Dimitrios Tsiachris, G Neil Thomas, Gregory Y H Lip, Konstantinos A Toulis

**Affiliations:** 1 Department of Endocrinology, 424 General Military Hospital, Thessaloniki, Greece; 2 Department of Cardiology, 424 General Military Hospital, Thessaloniki, Greece; 3 Athens Heart Center, Athens Medical Center, Athens, Greece; 4 3^rd^ Department of Internal Medicine, Aristotle University, Hypertension, Hypertension-24h ambulatory blood pressure monitoring center, Papageorgiou Hospital, Thessaloniki, Greece; 5 Institute of Metabolism and Systems Research, University of Birmingham, Birmingham, UK; 6 Laboratory of Hygiene, Social & Preventive Medicine and Medical Statistics, School of Medicine, Faculty of Health Sciences, Aristotle University of Thessaloniki, University Campus, Thessaloniki, Greece; 7 Institute of Applied Health Research, University of Birmingham, Birmingham, UK; 8 Liverpool Centre for Cardiovascular Science, University of Liverpool and Liverpool Heart & Chest Hospital, Liverpool, UK; 9 Department of Clinical Medicine, Aalborg University, Aalborg, Denmark

**Keywords:** differentiated thyroid cancer, atrial fibrillation, systematic review, meta-analysis

## Abstract

Differentiated thyroid cancer (DTC) represents the most common form of thyroid neoplasms and is becoming increasingly prevalent. Evidence suggests a possible relationship between DTC diagnosis and subsequent atrial fibrillation (AF). If confirmed, this may present an alarming health risk (AF) in an otherwise condition with a relatively good prognosis (DTC). The aim of this systematic review and meta-analysis is to provide for the first time a pooled estimate of AF incidence in DTC patients in comparison to healthy controls. A detailed search in electronic databases, clinical trial registries and grey literature was performed to identify studies reporting the incidence of AF in DTC patients. Newcastle–Ottawa quality assessment scale was used to assess study quality. We used a random effects (RE) generalized linear mixed model (GLMM) in pooling of individual studies and also calculated a prediction interval for the estimate of a new study. Six observational studies met the eligibility criteria, which included totally 187,754 patients with DTC and 199,770 healthy controls. The median follow-up period was 4.3 to 18.8 years; the incidence rate of AF was 4.86 (95% CI, 3.29 to 7.17, I^2^ = 96%) cases per 1000 person-years, while the incidence rate ratio was 1.54 (95% CI, 1.44 to 1.65, I^2^ = 0%, 95% PI, 1.33 to 1.78).This is the first meta-analysis to confirm that patients with DTC are at a high risk for developing AF, which may be attributed to a state of iatrogenic hyperthyroidism due to long-term thyrotropin suppression therapy.

## Introduction

Differentiated thyroid cancer (DTC), which includes papillary (85%) and follicular (12%) cancer, represents the most common form of malignant thyroid neoplasms. The age distribution is binomial (first peak at 35–40 years old, second peak over 70 years) with a consistent female preponderance (Rahbari* et al.* 2010, DeSantis* et al.* 2014). DTC is becoming increasingly prevalent, which is largely attributable to the increased detection of small papillary thyroid carcinomas and might be related to the widespread use of neck ultrasonography and/or cytological analysis of fine-needle aspirations (Davies & Welch 2014, Li* et al.* 2020). Thus, a timely diagnosis of the disease is common, so that an appropriate therapy is often instituted early, leading to a decrease in the disease-specific mortality rates since 1970s (https://www.cancerresearchuk.org/health-professional/cancer-statistics/statistics-by-cancer-type/thyroid-cancer/mortality; accessed (September) (2020)). In all, DTC has a favourable prognosis and, therefore, long-term survival is expected (DeSantis* et al.* 2014, Viola* et al.* 2016).

Total thyroidectomy is the cornerstone of initial management with or without adjuvant radioiodine-131 ablation (RAI). According to the European and American Thyroid Association, long-term levothyroxine suppression of thyrotropin (THST) is recommended in high- and selected intermediate-risk patients, whereas a low-normal thyroid-stimulating hormone (TSH) is considered to be the optimal therapeutic target for low-risk patients (Perros* et al.* 2014, Haugen* et al.* 2016). The rationale behind this approach is the minimization of potential TSH-mediated stimulation of tumour growth and the prevention of recurrence (Jonklaas* et al.* 2006, Brabant 2008, Goemann* et al.* 2017).

Over aggressive thyroxine (T4) therapy may lead to iatrogenic thyrotoxicosis, thus increasing the risk of atrial fibrillation (AF), mostly by increasing the heart rate (positive chronotropic effect) and conduction velocity (positive dromotropic effect). Impaired ventricular relaxation, the elevation of left atrial pressure due to increased left ventricular mass, ischaemia resulting from increased resting heart rate and re-entry might also be implicated in the development of AF (Shargorodsky* et al.* 2006, Bielecka-Dabrowa* et al.* 2009, Biondi & Cooper 2010, Abdulrahman* et al.* 2011). In patients with endogenous hyperthyroidism (e.g. Graves’ disease), low TSH levels are strongly related to AF risk (Camm* et al.* 2010). Therefore, it is reasonable to speculate that a similar relationship might exist in exogenous hyperthyroidism due to aggressive THST. On the other hand, endogenous and iatrogenic thyrotoxicosis should not be considered as identical clinical states in terms of circulating thyroid hormones since free triiodothyronine (T3) levels (the ligand to nuclear thyroid receptors in the cell) differ between the two states. Different T3/T4 ratios might also be associated with different health outcomes (Biondi & Cooper 2008).

In any case, if a high AF risk is confirmed in patients treated for DTC, then this might also increase the risk of stroke, heart failure and overall cardiovascular morbidity/mortality, and could be of major concern, considering the favourable long-term prognosis of DTC (Biondi & Cooper 2010, DeSantis* et al.* 2014, Carhill* et al.* 2015). Furthermore, RAI and in specific total cumulative dose might also be associated with the incidence of AF (Klein Hesselink* et al.* 2015). In other words, two independent predisposing factors to AF risk might be present in the management of DTC patients, while little is known about the magnitude of the effect (if any) and/or the individual patients’ characteristics that might affect this risk.

To this end, we performed a systematic review and meta-analysis to assess for the first time the incidence of AF in DTC patients and collate it in reference to healthy controls.

## Methods

The present systematic review followed the Preferred Reporting Items for Systematic reviews and Meta-Analyses (PRISMA) statement (Liberati* et al.* 2009). All research was conducted according to a protocol registered in OSF (Open Science Framework), available at: https://doi.org/10.17605/OSF.IO/YPCW7.

### Literature search strategy and study selection

The bibliographic search (detailed in Table 1) was performed in the electronic databases Medline (via the PubMed platform), the Cochrane Central Register of Controlled Trials (CENTRAL) and clinicaltrials.gov. A basic search strategy was developed for PubMed and modified accordingly for other research engines. We also searched PROSPERO to check if any similar meta-analysis is in progress in order to avoid duplication with our study. Conference abstracts and references of relevant studies and systematic reviews were perused and experts were contacted in order to identify unpublished studies.

**Table 1:** MEDLINE search strategy.*

**Search terms**	**Results**
1. Arrhythmia (All Fields)	240,499
2. Atrial fibrillation (All Fields)	77,853
3. 1 OR 2	259,607
4. Thyroid cancer (All Fields)	75,780
5. Thyroid neoplasm (All Fields)	67,461
6. Thyroid carcinoma (All Fields)	71,642
7. 4 OR 5 OR 6	78,956
8. Exp animals/not humans.sh.	263,760
9. 3 AND 7 NOT 8	187
10. Atrial fibrillation (Mesh)	55,914
11. Thyroid neoplasms (Mesh)	52,823
12. 10 AND 11	25

*Via Pubmed, performed on 5 December 2019.

Records retrieved from the search were imported in reference management software. After removing the duplicate records, two reviewers (GK and ID) screened titles and abstracts independently and full texts were investigated for eligible studies. Differences in opinion between the two reviewers were resolved by a third reviewer (CA).

### Eligibility and exclusion criteria

**Figure 1:**
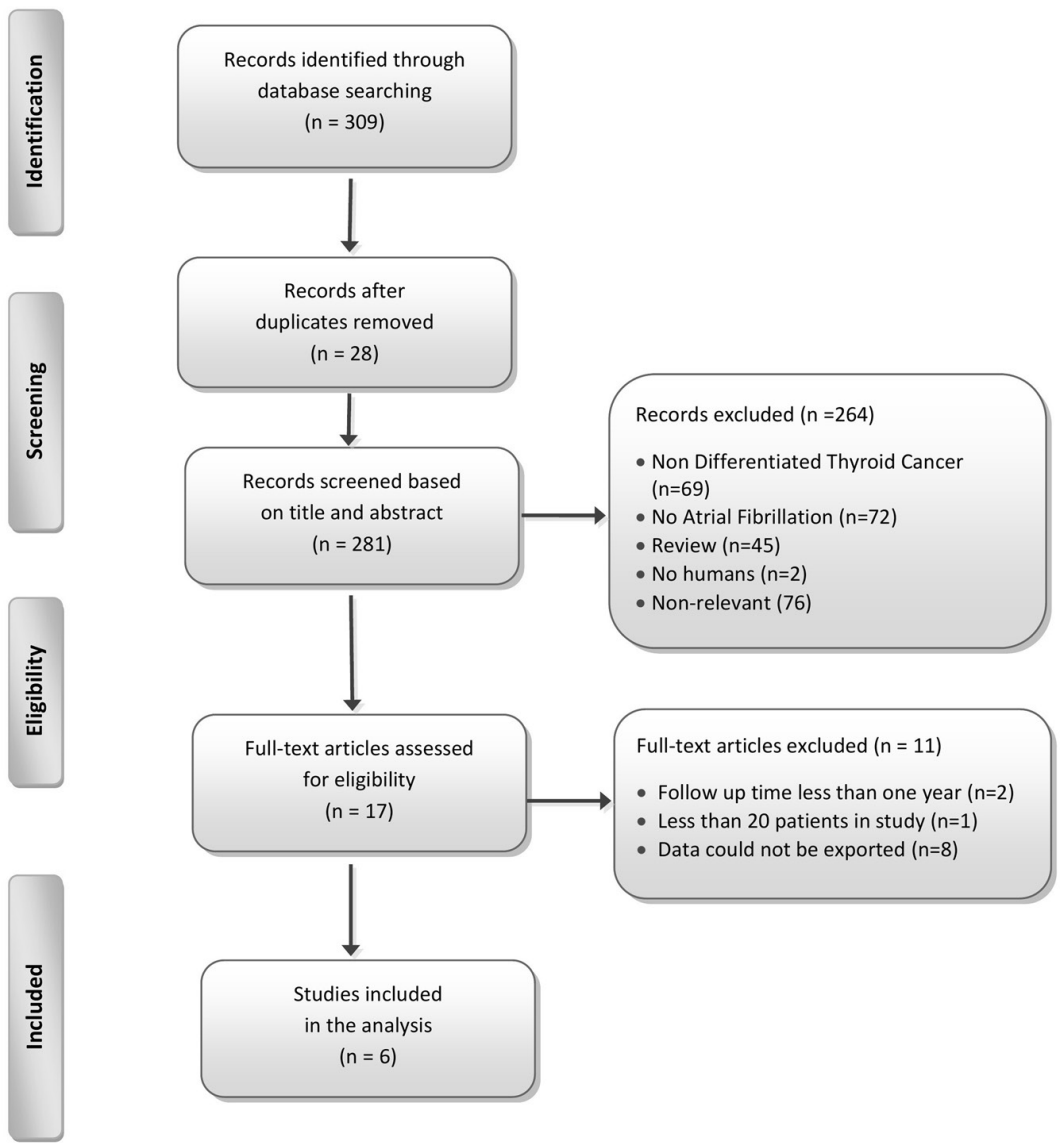
Summary of evidence search and selection.

We included original case-control and cohort studies reporting estimates of the risk of incident AF in patients with DTC. There was no limitation in publication date or language. Pre-defined exclusion criteria were applied to the following studies: (i) with no original data (review articles, commentaries, editorials) or of case-series design, (ii) not reporting on incident AF rates, (iii) with less than 20 individuals, (iv) with unknown or less than 1-year duration of follow-up, (v) with no accessible or extractable data. A detailed summary of the excluded studies is presented in the flow chart (Fig. 1).

### Data extraction and quality assessment in individual studies

Two reviewers (G K, I D) examined the search results and screened the titles and abstracts. Subsequently, full texts of the selected studies were obtained and evaluated independently by the two reviewers (G K, I D) for eligibility. Any discordance regarding study eligibility was resolved by consultation with a senior author (K T). When necessary, we attempted to contact the study authors to clarify uncertainties in the study design or results.

Two reviewers (G K, I D) assessed the potential risk of bias in the included studies using the version of the Newcastle–Ottawa quality assessment scale (NOS) developed for case-control studies. On the basis of NOS quality assessment scale, a maximum of nine points was assigned for the least risk of bias in three domains: (i) selection of study groups (four points); (ii) comparability of groups (two points); (iii) ascertainment of exposure (three points) for case-control studies (http://www.ohri.ca/programs/clinical_epidemiology/oxford.asp). Any disagreement was resolved by discussion or by the involvement of a senior investigator (K T).

### Data synthesis and statistics

The primary outcome was the aggregate incidence rate (IR) of AF in patients with DTC. Following Stijnen *et al.,* we used a random effects (RE) generalized linear mixed model (GLMM) in pooling of individual studies (Stijnen* et al.* 2010, Schwarzer & Chemaitelly 2019). Data referring to matched, cancer-free, control patients were also considered for quantitative synthesis. A pooled incidence rate ratio (IRR) estimate was calculated using a random effects GLMM model, as well. A prediction interval (PI) for the estimate of a new study was also calculated as proposed by Higgins (Higgins* et al.* 2009).The total number of person-years at risk, as reported by individual studies, was used. When these were not reported, the average length of follow-up and the total number of patients at the end of the study were used to approximate the total person-years (Pedder* et al.* 2016). In addition, to investigate sources of potential heterogeneity and to test the robustness of our results, a sensitivity analysis, incorporating studies with the highest quality, was conducted. A score of 9 points in the NOS quality assessment scale indicated a highest quality study. Analysis was performed in R v3.6.0 utilizing the meta package (Schwarzer 2007)

## Results

### Search results and characteristics of the eligible studies

The systematic literature search identified 281 potentially eligible articles; 6 studies finally met the eligibility criteria and were included in the review (Fig. 1). All included studies were observational in design and were conducted in Canada, the Netherlands, Finland, Korea, the United States and the United Kingdom between 2012 and 2019. Studies and patient characteristics are summarized in Table 2. Briefly, a total of 387,524 people were included in the studies of the meta-analysis: 187,754 patients with DTC and 199,770 healthy, cancer-free, matched controls with a follow-up time (mean or median) ranging from 4.3 to 19 years. The NOS scores of the studies were above 5 and presented in Table 3.

**Table 2:** Summary of included studies, individual study details and findings.

Author, year	Country	Patients (DTC)							Controls				TSH	Outcomes	Significant positive results vs controls
		*n*	Age (mean ± s.d., years)	Gender F/M (%)	Total thyroidectomy	RAI	Follow-up time (mean or median,years)	TSH (median, mIU/L)	*n*	Age (mean ± s.d.,years)	Gender F/M (%)	Follow up-time (mean or median,years)			
Abonowara *et al*. 2012	Canada	136	52.0 ± 15.8	85	All	NR	11*	0.17	No control group	–	–	–	–	Prevalence of AF	Prevalence of AF higher than general population AF higher in ages over 60. No correlation with level of TSH suppression.
Klein Hesselink *et al.* 2015	Netherlands	518	48.6 ± 14.0	74.7	All	All	8.7	0.10	1563 (matched for age, sex, without DMT1, proteinuria and kidney failure, no data for endogenous thyroid disorders)	48.6 ± 13.4	74.5	10.6	NR	Incident AF	2.5-fold increased risk of AF in DTC patients independent from AF risk factors. Higher risk in older and hypertensive. Higher cumulative dose of RAI associated with increased AF risk. No relation between TSH level and incident AF.
Pajamaki *et al*. 2018	Finland	901	48.8 ± 15.9	81	78%	81%	18.8	0.11	4485 (matched for age, sex, residency, no data for endogenous thyroid disorders)	48.7 ± 15.8	81	19	NR	Morbidity due to any CVD	Morbidity due to any CVD and risk of AF higher in DTC patients than controls.
Suh *et al.* 2019	Korea	182,419	47 ± 11.3	84.2	All	57.3%	4.3*	NR	182,419 (propensity score for age, sex, residence, insurance level, disability, hypertension,diabetes dyslipdemia, participants received Lt4 1 year prior index date excluded)	47 ± 11.3	84.2	4.3*	NR	Incidence of CHD, ischemic stroke	DTC patients without previous history of CVD higher risk of CHD and stroke. AF risk dose dependent, small proportions of stroke incidence
Toulis *et al.* 2019	UK	3009	50.7 ± 16.1	76	All	NR	4.8	NR	11,303 (matched for age, sex, BMI, smoking, subset treated for hypothyroidism)	50.4 ± 15.7	76.2	5.4	NR	Risks of circulatory disease.	1.7 fold increase in risk of AF in DTC patients in the absence of CVD disease.
Wang *et al.* 2015	USA	771	48 ± 14	73.8	All	75 (TSH <0.4)– 60% (TSH >0.4)	6.5	NR	One cohort divided (TSH <0.4 mIU/L and >0.4 mIU/L)	–	–	–	–	Recurrence AF risk Osteoporosis risk	No difference in AF risk between two groups. Increased risk of incident osteoporosis in the suppressed group (TSH <0.4 mIU/L ).

*Mean follow-up time

AF, atrial fibrillation; CHD, coronary heart disease; CVD, cardiovascular disease; DTC, differentiated thyroid cancer; DMT1, diabetes mellitus type 1; F, female; Lt4, levothyroxine; M, male; N, number; NR, not reported; RAI, radioactive iodine therapy; TSH, thyroid stimulation hormone.

**Table 3:** Detailed Newcastle–Ottawa score (NOS) grading for each study.

Author,year	Selection	Comparability	Outcome	Total
Abonowara *et al*. 2012	2	1	3	6
Klein Hesselink *et al.* 2015	4	2	3	9
Pajamaki *et al.* 2018	4	2	3	9
Suh *et al.* 2019	3	2	3	8
Toulis *et al.* 2019	4	2	3	9
Wang *et al.* 2015	3	1	3	7

### Systematic review

Four studies reported that the risk of incident AF was significantly higher in DTC patients compared to cancer-free controls, adjusted for established risk factors, such as age, hypertension, diabetes and coronary artery disease (Klein Hesselink* et al.* 2015, Pajamäki* et al.* 2018, Suh* et al.* 2019, Toulis* et al.* 2019), whereas another study reported a higher AF prevalence when compared to the general population (Abonowara* et al.* 2012). The sixth study included a cohort of DTC patients with low- and intermediate-risk for recurrence, examining the benefits of TSH suppression and potential skeletal and cardiovascular adverse events (Wang* et al.* 2015). In this study, the rate of AF was also reported. Overall, the IR for AF in DTC patients ranged from 2.2 to 9.36 per 1000 person-years; in those studies which included healthy controls, the adjusted hazard ratio (aHR) for AF in the DTC cohort ranged from 1.29 to 2.5.

None of the included studies could provide evidence for a possible association between the aggressiveness of THST and the risk for AF, although indirect evidence was reported. Suh * et al.* reported that DTC patients were consistently found to be at a higher risk of developing AF across all categories of levothyroxine dosage, but the aHR was found to be highest in the highest dosage quartile, which might be indicative of a dose-response relationship (Suh* et al.* 2019). Of note, no correction for body weight was available. Finally, Pajamaki *et al.* reported that patients with TSH <0.1 mU/L had an increased risk (HR: 1.27, CI:1.03–1.58) of cardiovascular morbidity mostly attributed to AF (Pajamäki* et al.* 2018).

The relation between RAI and incident AF was explored in a single study. Klein *et al.* reported that cumulative radioiodine dose might be correlated with a higher AF risk independently of TSH levels (Klein Hesselink* et al.* 2015). A subgroup analysis focusing on the patients treated with adjuvant RAI was also planned, but not conducted, because data regarding incident AF in this cohort were not available.

In patients with DTC, the risk of AF was related significantly with older age and hypertension in two studies (Abonowara* et al.* 2012, Klein Hesselink* et al.* 2015). On the contrary, a large retrospective cohort study with 6900 Swedish DTC patients reported that DTC patients were at a higher risk of hospitalization for AF, especially those who were younger men and during the first 5 years of the follow-up time (Zoltek* et al.* 2020). This study was not included in our systematic review and meta-analysis as the primary outcome was the risk of hospitalization for AF and other cardiovascular endpoints. Only two studies involved patients without pre-existing circulatory disease, while one study was limited to low- and intermediate-risk patients (Wang* et al.* 2015, Suh* et al.* 2019, Toulis* et al.* 2019). Table 4 presents the event rate (AF) in patients with DTC and in healthy controls.

**Table 4:** Number of events, person-years and IR of DTC patients and controls in the eligible studies.

Study	DTC patients	Follow-up time (mean/median, years)	Number of events	Person-years	IR (per 1000 person-years)	Controls	Follow-up time (mean/median, years)	Number of events	Person-years	IR (per 1000 person-years)	HR (adjusted, model)
Abonowara *et al.* 2012*+*	136	11	14	1496*	9.36	–	–	–	–	–	–
Klein Hesselink *et al.* 2015	518	8.7	35	5646	6.20	1563	10.6	42	15,357	2.73	2.47 (1.55–3.95) Fine/gray regression model
Pajamaki *et al.* 2018	901	18.8	120	16,939*	7.08	4485	19	485	85,215*	5.69	1.29 (1.06–1.57) Cox model
Suh *et al.* 2019	182,419	4.3	1737	789,756.1	2.20	182,419	4.3	1113	796,490.2	1.4	1.43 (1.27–1.62)^1^, 1.41 (1.26–1.58)^2^, 1.66 (1.49–1.85)^3^, 1.78 (1.60–1.98)^4^ Cox model
Toulis *et al.* 2019	3009	5	102	19,345.9	5.27	11,303	5.4	262	77,781.7	3.37	1.71 (1.36–2.15) Cox model
Wang *et al*. 2015*+*	771	6.5	17	5,012*	3.39	–	–	–	–	–	–

*Calculated person-years, ^1^Group Lt4 dose of <115 mcg, ^2^Group Lt4 dose of 115–144 mcg, ^3^Group LT4 dose of 145–169 mcg, ^4^Group LT4 dose of >170 mcg, ^+^Studies without control group.

DTC, differentiated thyroid cancer; HR, hazard ratio for atrial fibrillation in studies with control group; IR, incidence rate.

### Meta-analysis

**Figure 2:**
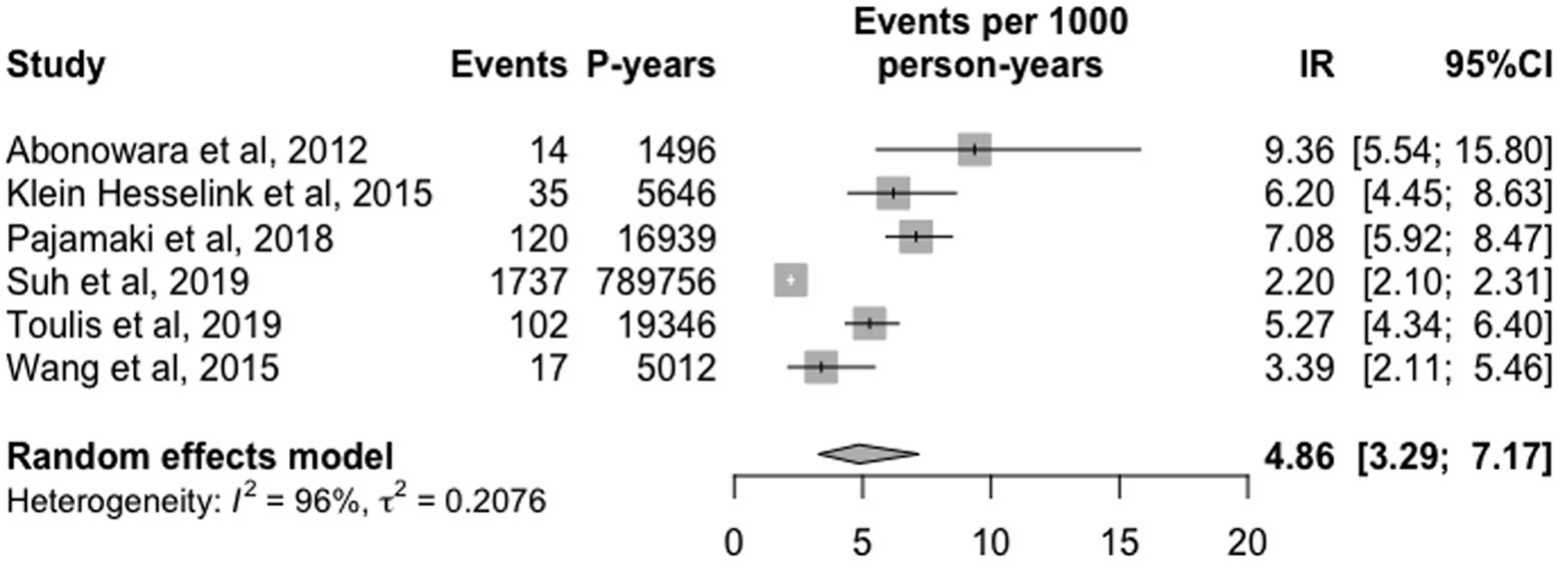
Forest plot of aggregate IR of AF in patients with DTC.

**Figure 3:**
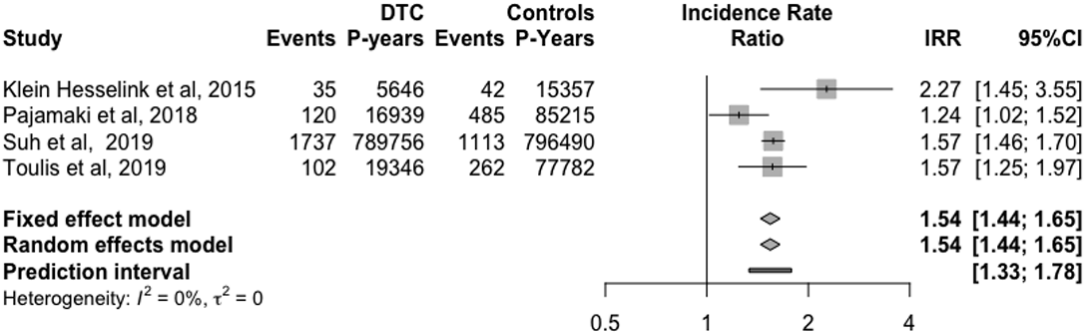
Forest plot of pooled IRR of AF in patients with DTC and their healthy, cancer-free controls

The overall IR of AF was estimated to be 4.86 (95% CI, 3.29 to 7.17, I^2^ = 96%, RE) cases per 1000 person-years (Fig. 2). Additionally, the pooled IRR of AF was equal to 1.54 (95%CI, 1.44 to 1.65, I^2^ = 0%, 95%PI, 1.33 to 1.78, RE) (Fig. 3). To further explore and address the issue of high heterogeneity among studies in our meta-analysis (I^2^ = 96%), a sensitivity analysis was undertaken including the highest quality observational studies (NOS = 9) (Klein Hesselink* et al.* 2015, Pajamäki* et al.* 2018, Toulis* et al.* 2019). In this analysis, incident AF was found to be 6.13 cases per 1000 person-years (95% CI, 5.19 to 7.23, I^2^ = 40%, RE), while the IRR of AF was 1.49 (95%CI, 1.17 to 1.89, I^2^ =34%, RE).

## Discussion

In this study, the incidence of AF in patients with DTC by meta-analysis was found to be 4.86 cases per 1000 person-years. This estimate was based on studies with an adequate follow-up duration (mean follow-up ≥ 4.3 years), considering that aggressive suppression therapy, when needed, is usually applied in the first 5 years after DTC diagnosis. To further quantify the magnitude of difference between patients with DTC and their healthy counterparts, we provided an IRR approximation, incorporating in parallel variations in the length of follow-up among studies. Our findings suggest that patients with DTC are expected to be at a higher risk for AF, namely about 50%, compared to healthy controls (IRR =1.54).

In line with the European (Perros* et al.* 2014) and American guidelines (Haugen* et al.* 2016), long-term levothyroxine suppression following total thyroidectomy with or without adjuvant RAI is the recommended approach in patients with high-risk DTC. This intervention, aimed to prevent tumor recurrence, especially in the high and intermediate-risk group, leads to a state of iatrogenic hyperthyroidism. Endogenous hyperthyroidism is a well-known risk factor for AF, ischaemic stroke, angina, myocardial infarction and congestive heart failure in patients with underlying cardiovascular disease (Sawin* et al.* 1994, Ertek & Cicero 2013). Similarly, overt and subclinical exogenous hyperthyroidism may also be associated with cardiovascular disease, including AF, diastolic dysfunction, and left ventricular hypertrophy (Shargorodsky* et al.* 2006, Biondi & Cooper 2010, Abdulrahman* et al.* 2011).

In a meta-analysis of 8711 participants from 5 cohorts with a mean follow-up of 8.8 years, the estimated incidence of AF in patients with subclinical hyperthyroidism was 17.1 cases per 1000 person-years (Collet* et al.* 2012). Furthermore, Selmer* et al.* (2012) reported that incident AF in patients with overt and subclinical endogenous hyperthyroidism was 12.5 cases and 8.4 cases per 1000 person-years, respectively, based on a study population of 586,460 individuals. Finally, another large population-based study from Norway (932,913 people) calculated that incident AF was ~35.0 cases per 1000 person-years within 3 months after the diagnosis of endogenous hyperthyroidism, declining to ~9.3 within 3 years of follow-up (Dekkers* et al.* 2017). The above findings suggest that the risk of AF is consistently elevated across different types of hyperthyroidism.

Patients with endogenous thyroid disease and a suppressed TSH are at a higher risk for AF. It is reasonable to anticipate a similar relationship in states of exogenous hyperthyroidism due to aggressive THST. Surprisingly, none of the included studies could demonstrate an association with AF and lower TSH values, either because of the sample size (Abonowara* et al.* 2012), study design and number of events (Wang* et al.* 2015) or different TSH assays (Klein Hesselink* et al.* 2015). Another possible explanation is that many of these studies only analyse a single TSH measurement at single specific point-time during the patient follow-up, which is an inadequate measure of ongoing thyroid hormone status. Similarly, the single observation that total cumulative radioiodine dose might be an independent risk factor for AF has not been replicated.

Despite the observation that all the studies including controls reported a consistent increase in AF risk in patients with DTC relative to healthy counterparts, a notable variation in the reported IRs was observed, ranging from 2.2 to 7.08 in the DTC cohorts. This may be attributed to high heterogeneity (between-study variation) regarding baseline characteristics of the included population, sample size, follow-up time, definition and diagnosis of AF (paroxysmal, persistent, permanent, atrial flutter– ICD codes or 24 h Holter) and the aggressiveness of TSH suppression. More specifically, the lowest incidence rate was observed in a DTC cohort from Korea, which is in line with epidemiologic data regarding the global burden of AF (Chugh* et al.* 2014). Regional heterogeneity in the epidemiology of AF has been observed and reported in the literature and specifically, in Asia, lower AF incidence rates have been reported either because of sociodemographic factors or other factors (Chugh* et al.* 2014).

In addition, AF is associated with an increased risk of thromboembolic disease and therefore an increased incidence of ischemic cerebrovascular disease (Chugh* et al.* 2014). Since DTC patients are more likely to be diagnosed with AF, it is plausible to assume that there is a similar tendency in incident stroke. However, the reports from two studies are conflicting. Toulis* et al.* (2019), found that DTC cohort had a modest risk increase for stroke or transient ischaemic attack, while Suh* et al.* (2019) found that a small number of ischaemic stroke events in the DTC patients were related to AF, although they were at a 2.5-fold risk for other incident cerebrovascular disease.

From a clinical point of view, besides stroke prevention (prophylactic use of anticoagulants) a discrete approach might warrant further consideration in patients with iatrogenic hyperthyroidism regarding rhythm and rate control. Although there is no definite evidence that beta-blockers can prevent AF in subclinical hyperthyroidism, they may offer a reasonable option as a rate control intervention independently of the underlying disease, especially in patients with accompanying hypertension and heart failure, conditions that minimize Ic antiarrhythmics administration. On the other hand, amiodarone, an iodine-rich drug with a half-life of 100 days, is not recommended in those patients with DTC for whom RAI therapy is indicated since amiodarone interferes with ^131^ Iodine uptake for a long time after discontinuation and thus, RAI therapy or imaging is postponed if the patient has been treated with amiodarone within the previous year (Perros* et al.* 2014).

### Strengths and limitations

To the best of our knowledge, this is the first attempt to provide an evidence-based estimate regarding the long-term effects of DTC treatment on the risk of AF. The strengths of our study include the comprehensive systematic search and the strict methodology regarding the identification of relevant studies and the selection process. Studies with unclear methodology or short follow-up time were excluded. On the other hand, we acknowledge that our meta-analysis had several limitations, including the observational and retrospective nature of the included studies, the high heterogeneity in study populations and baseline characteristics, the lack of data on the RAI cohort, thyroid function tests and discrepancies in AF definition and TSH assays. Such heterogeneity in population characteristics should be interpreted with caution in the context of generalizing the results of the present study to other settings. The results of our meta-analysis were largely attributable to the study conducted in Korea (Suh* et al.* 2019), which included over 350,000 participants. However, even with the exclusion of this study from the sensitivity analysis, the incidence of AF in patients with DTC stills remains significantly higher compared to controls. Moreover, lack of data regarding the histopathological type or disease stage and most importantly, the degree and aggressiveness of TSH suppression prevented any attempt to explore an association either of TSH suppression or cumulative RAI dose and development of AF and ischaemic cerebrovascular disease. Finally, since several AF cases are asymptomatic and, in some studies, the data were derived from primary care or claim databases, in which a diagnosis is matched to an ICD-10 code, the possibility that the true overall incidence rate might be underestimated cannot be precluded.

## Conclusion

This is the first meta-analysis to confirm that patients with DTC are at a high risk for developing AF, which may be attributed to a state of iatrogenic hyperthyroidism due to thyrotropin suppression therapy. Hence, periodical screening for AF and its complications in selected cases might be considered.

## Declarations of interest

All authors declare that there is no conflict of interest that could be perceived as prejudicing the impartiality of the research reported.

## Funding

This research did not receive any specific grant from any funding agency in the public, commercial or not-for-profit sector.

## Author contribution statement

G K: conceptualized the study design, performed the literature search, critically appraised the articles as first independent reviewer and formulated the paper. I D: conceptualized the study design, performed the literature search, critically appraised the articles as second independent reviewer, performed the statistical analysis. C A: critically appraised the paper, third independent reviewer. E B: performed the statistical analysis, critically appraised the paper. K N, D T, N T, G L: critically appraised the paper and made final suggestions. K T: conceived the idea, conceptualized the study design, supervised the statistical analysis, critically appraised the paper and made final suggestions. All authors approved the final version of the manuscript prior to submission.
